# Pediatric heart transplant survival in single ventricle disease

**DOI:** 10.1016/j.jhlto.2025.100396

**Published:** 2025-09-24

**Authors:** Jillian Wen, Marc Richmond

**Affiliations:** Division of Pediatric Cardiology, Morgan Stanely Children’s Hospital, Columbia University Irving Medical Center, New York, NY

**Keywords:** Pediatric heart transplantation, Single ventricle, Fontan, Outcomes, Mortality

## Abstract

Outcomes in pediatric heart transplantation have steadily improved since the first pediatric heart transplant was performed by Dr. Kantrowitz and his team in 1967; however, there is still progress to be made. Patients with congenital heart diseases (CHD) consistently have worse waitlist mortality, post-transplant survival, infection rates, and post-transplant lymphoproliferative disease (PTLD) risk, with single ventricle disease (SVD) patients being at particularly high risk for rejection and rejection with severe hemodynamic compromise (RSHC). Furthermore, there has been little improvement in the development of cardiac allograft vasculopathy (CAV) and PTLD over the decades, and survival following diagnosis continues to remain poor for all patients regardless of diagnosis. Hopefully, as new advances in immunosuppression, mechanical support, and surgical techniques emerge, we will continue to develop a deeper understanding of the co-morbidities associated with pediatric heart transplant to further improve the survival and quality of life of pediatric heart transplant recipients.

## Background

Single ventricle heart disease (SVD) encompasses a complex and heterogeneous group of congenital heart diseases (CHD) in which patients have only one functional ventricle to adequately provide either systemic or pulmonary circulation. There are many subtypes of SVD, most commonly hypoplastic left heart syndrome (HLHS), but also unbalanced atrioventricular canal defects, double inlet left ventricle, tricuspid atresia, certain double outlet right ventricle subtypes, and severe Ebstein anomaly. SVD accounts for approximately 7% of all CHD with an incidence of 4-8 per 100,000 live births.^1^

The physiology of SVD is characterized by parallel systemic and pulmonary circulations requiring staged surgical palliation to separate the two circulations while optimizing oxygenation and reducing ventricular volume load. This is achieved through a series of surgical procedures to establish the single working ventricle as the sub-systemic ventricle and to create a pathway for passive pulmonary blood flow. The first stage in palliation is typically performed within the first week of life, whereby the single working ventricle is used to establish reliable systemic circulation, and a systemic-to-pulmonary shunt is created to provide pulmonary blood flow.^2^ The next palliative stage is the Glenn procedure, ideally performed at 3-6 months of life, where a superior cavopulmonary anastomosis is created to allow for passive blood flow from the upper body to the lungs, oftentimes allowing for the removal of the systemic to pulmonary shunt.^3^ The final stage in palliation is the Fontan procedure, typically performed during toddler years, where a total cavopulmonary connection is established by incorporating the inferior vena cava into the pulmonic circulation, thus completing the separation of the systemic and pulmonary circulations.^4^

While this palliative approach has significantly improved survival in the single ventricle population and is now the standard of care for patients with SVD, the altered physiology following each stage in repair creates unique challenges that may eventually require consideration for heart transplantation. As the final stage in palliation, Fontan circulation creates an especially interesting physiology and deserves special consideration. Due to the lack of a sub-pulmonary ventricle and resultant passive blood flow to the lungs, the pulmonary and systemic vasculature undergo chronic changes that can ultimately lead to the intolerance of the Fontan physiology, often referred to as Fontan failure.^5^, ^6^, ^7^ Fontan failure is a heterogenous process that can occur in the presence or absence ventricular dysfunction with a wide spectrum of clinical manifestations, including failure to thrive, exercise intolerance, fluid overload, liver disease, kidney disease, and lymphatic dysfunction (most notably protein-losing enteropathy (PLE) and plastic bronchitis (PB)).^5^, ^6^, ^8^, ^9^ Progression of Fontan failure often persists despite aggressive medical management, with heart transplant being the only definitive cure and Fontan failure being one of the largest indications for heart transplant in the modern era.^10^

According to the Pediatric Heart Transplant Society (PHTS) and International Society for Heart and Lung Transplant registry data, although cardiomyopathy remains the most frequent indication for pediatric heart transplant, more and more children are being transplanted for CHD.^11^ Within the CHD population, the proportion of children listed for transplant due to SVD has varied across the years but is higher now when compared to prior eras. In the early years of pediatric heart transplant, there was a focus on improving the short-term outcomes, including early survival and rejection, especially in the complex SVD patient population. However, as surgical techniques, immunosuppression, and overall survival have continued to improve, there has been a contemporary shift in focus from short-term to long-term outcomes, including longer-term survival as well as post-transplant complications such as late rejection, infection, renal disease, malignancy, and cardiac allograft vasculopathy (CAV). This article seeks to review the historical trends and current outcomes in pediatric patients with SVD following heart transplant.

## Waitlist mortality

Although the waitlist mortality of pediatric patients awaiting heart transplant has improved over the last 3 decades, it remains unacceptably high, with an estimated 13%-15% of children dying on the waitlist in the most recent era.^12^, ^13^ Unfortunately, this number is even higher within the CHD population.^12^, ^13^, ^14^ A report by Richmond et al reviewing the last three decades of PHTS registry data showed that the 6-month waitlist survival for patients with CHD is only 80% in the most recent decade, compared to 88% in patients with cardiomyopathy.^13^

Interestingly, within the CHD population, those patients with SVD have better waitlist survival than patients with biventricular circulation (74% vs 64% at 12 months).^13^ Differences in these populations are apparent, as SVD patients are overall older at the time of listing, more often a lower priority listing status, and are less frequently on mechanical ventilatory or circulatory support.^13^ However, they are also more likely to have longer median waitlist times.^13^, ^15^ Furthermore, waitlist survival amongst patients with SVD differs by palliative stage. In the most recent decade, infants listed prior to surgical palliation (stage 0) and those at stage I palliation have the worst waitlist outcomes, whereas patients listed after stage III palliation (Fontan failure) have the best.^13^ A recent large retrospective cohort study examining transplant survival in patients with Fontan failure reported a waitlist mortality of only 5.9% and found several factors associated with death on the waitlist, including higher New York Heart Association class (which may be exacerbated by higher aortopulmonary collateral burden and lead to more hospitalizations, both of which were also identified as risk factors), younger age, sleep apnea, non-enrollment in school/work (another possible sequela of higher New York Heart Association class), and single-parent home.^16^

The use of ventricular assist devices (VADs) has, on the whole, significantly improved the landscape of waitlist outcomes, but the complexities of using VADs in children with CHD likely contributes to the discrepancy in waitlist mortality between patients with and without CHD. Even for those CHD patients who receive a VAD, waitlist outcomes are inferior to non-CHD children with a VAD.^14^, ^17^, ^18^, ^19^ Even within the VAD-CHD population, there are disparate outcomes based on physiology and stage of SVD palliation, with SVD patients placed on VAD as stage I or stage II palliation at significantly higher risk of mortality when compared to SVD patients at stage III palliation.^18^, ^19^ This highlights a need to further explore the risk factors for VAD-SVD patients prior to Fontan palliation, as well as a need for better mechanical support options in young infants and those with complex CHD.

## Survival outcomes

Overall post-transplant survival outcomes have improved over the decades for all patients regardless of pre-transplant diagnosis, including those with SVD.^11^, ^13^, ^15^, ^20^ However, patients transplanted for CHD continue to have worse short and long-term post-transplant survival compared with patients transplanted for cardiomyopathy, although with comparable survival between CHD-SVD and CHD-biventricular disease (BVD) patients.^11^, ^13^, ^15^, ^20^

In last year’s PHTS Registry report, overall survival was not significantly different when stratified by palliative stage in SVD, although some interesting trends arose when those patients were further stratified by era (with era one spanning 1993-2004, era two 2005-2009, and era three 2010-2019).^13^ During era one, about 50% of SVD patients were listed at stage 0 and 21% listed after stage three. Although the babies listed at stage 0 had worse waitlist survival, they had the best post-transplant survival. Conversely, in era two, significantly fewer patients were transplanted at stage 0 and survival for those patients was also worse. This trend reflects a shift in the surgical management in patients with SVD (particularly in HLHS), where primary heart transplant was frequently considered before staged repair during era one but became less and less frequent as surgical techniques and outcomes improved for staged repair during era two. By era three, survival had improved with comparable survival across all palliative stages (∼80%-90% at 5 years post-transplant).

Interestingly, the most recent decade saw an increase in transplants at stage I and a corresponding decrease in transplants at stage II. However, some other large-volume single-center studies have reported the opposite, with more patients listed at stage II of palliation than at stage I.^21^, ^22^, ^23^ This discrepancy likely represents the variability in managing stage I patients with complex and higher-risk physiology.^21^, ^22^, ^23^ This study also demonstrated a four-fold increase in patients transplanted following stage III from era one to era three, consistent with projections that the need for transplant in the Fontan patient population will continue to grow in the coming decades.^5^, ^7^

There are limited data examining risk factors for mortality following heart transplant specifically in the SVD population, although more generalized studies have demonstrated an association between the presence of CHD and the number of prior sternotomies with increased mortality, both of which are relevant to the single ventricle population.^15^, ^20^, ^24^ A prior study using the PHTS database to examine outcomes following pediatric heart transplant in patients with HLHS and the Norwood procedure identified African American race as a risk factor for post-transplant mortality, an association found in other non-SVD specific studies as well.^21^ Interestingly, in this report, United Network for Organ Sharing listing status, age, and the presence of panel reactive antibodies (PRAs) >10% did not have any significant associations with mortality following transplant.

Within the Fontan population, the presence of pre-transplant renal dysfunction and PB have been found to be associated with early mortality post-transplant, though long-term survival in patients with PB is comparable to survival of those without.^5^, ^15^, ^25^ Other factors that have been associated with increased 1-year post-transplant mortality in this population include lower oxygen saturations, Fontan pathway or pulmonary artery obstruction, higher class II PRAs, higher grade portal varices, and the presence of a mental health condition requiring treatment.^16^ Additionally, some studies have reported increased mortality in patients who were transplanted <6 months following Fontan operation, emphasizing the importance of achieving stable single ventricle physiology, whether through VAD placement, Fontan takedown with reversion to Glenn physiology, or other means.^15^, ^26^ Interestingly, PLE does not appear to increase waitlist mortality or post-transplant mortality independently; however, many patients with PLE experience poor nutrition and growth, which is a known risk factor for worse outcomes in CHD following transplant.^16^, ^27^

## Complications

### Rejection

With advances in induction and maintenance immunosuppression techniques, the overall incidence of rejection after pediatric heart transplant has declined over the decades.^11^, ^20^ However, rejection continues to remain a major cause of morbidity and mortality following heart transplant and is a major risk factor for poor outcomes, including CAV, retransplant, and death.^24^, ^28^ Rejection with severe hemodynamic compromise (RSHC), defined as an episode of rejection requiring the use of intravenous inotropes for graft function support, is also an important outcome to consider, as graft survival following RSHC is poor.^13^ Similar to overall rejection, RSHC has decreased over the decades; however, survival after RSHC has not changed over time irrespective of pre-transplant cardiac diagnosis and remains poor, with a 63% 5-year survival following an episode of RSHC.^13^

When comparing the SVD and BVD populations, patients with SVD have lower freedom from rejection as well as a lower freedom from RSHC (88% in CHD-BVD vs 78% in CHD-SVD at 5 years).^13^ The difference in freedom from rejection in SVD vs BVD patients appears to be most significant in the early phase (<1 year following transplant), which is particularly salient when considering post-transplant outcomes, as many studies have indicated that patients with rejection treated within their first year of transplant have decreased long-term survival.^11^, ^13^, ^29^

Rejection outcomes in Fontan patients is of particular interest, as these patients are often human leukocyte antigen-allosensitized due to the significant exposure to blood products and homograft materials over the course of multiple surgeries, and many have altered immune systems due to PLE and other factors.^5^ High allosensitization has been associated with decreased one-year post-transplant survival in the overall pediatric heart transplant population, although some reports have suggested that the post-transplant outcomes in highly-sensitized Fontan patients may not be as significantly affected.^5^, ^30^, ^31^ Additionally, it may be difficult to characterize and measure how presensitized these patients are if they have significant immunoglobulin losses due to PLE. Some Fontan patients with PLE exhibit low PRAs and have low donor-specific antibodies (DSAs), which may be due to the gastric loss of immunoglobulins and antibodies; however, it’s unclear if restoration of gut function and resolution of PLE following transplant can lead to the development of newly detected DSAs. One single-center study involving a limited number of patients demonstrated that 50% of PLE patients developed new class II DSAs by one year following heart transplant compared to 30% in non-PLE patients, but the difference did not meet statistical significance.^32^ In this study, the PLE patients had higher rates of antibody-mediated rejection compared to non-PLE patients, despite no significant difference in newly detected DSAs.^32^

To add to the uniqueness and heterogeneity of the Fontan population, many patients will have lymphopenia with the selective loss of CD4+ helper T cells.^33^, ^34^ The alterations in the immunological environment may be further exacerbated by the loss of immunoglobulins in the gastric mucosa in PLE, although hypogammaglobulinemia may occur even in the absence of PLE.^33^, ^34^ Despite this, a recent study by Mantell et al examining the effects of different immunophenotypes in Fontan patients found that for the first 5 years post-transplant, there was no difference in the time to first episode of rejection or the number of rejection events per patient-year, irrespective of the presence of lymphopenia or PLE.^34^

### Infection

Although infection rates and infection-related mortality have declined over the decades, infection still remains a significant cause of post-transplant morbidity and mortality and was previously demonstrated to account for about 12% of deaths during the first year following transplant, with infants being at particularly high risk of more severe/chronic infections.^11^, ^13^, ^35^ Infection in the CHD population, and even more so the SVD population is of particular concern due to higher rates of human leukocyte antigen-allosensitization necessitating additional immunosuppression. Furthermore, the potential need for postoperative extracorporeal membrane oxygenation or delayed sternal closure places these patients at an even higher risk of life-threatening infection. Patients with CHD have lower freedom from infection compared to patients with cardiomyopathy, with no difference between CHD-SVD and CHD-BVD patients.^13^ In Fontan patients, those with a compromised immune system prior to transplant (i.e. lymphopenia and/or PLE) have a higher frequency of early infections as well as a higher number of total yearly infections compared to Fontan patients without immunocompromise, whose yearly infection rate was comparable to the CHD-BVD population.^34^ This difference in infection is most pronounced during the first year of transplant, which is consistent with reports that PLE and lymphopenia typically resolve by one year post-transplant.^27^, ^34^, ^36^ Interestingly, in the PHTS study examining Fontan immunophenotypes, viral pathogens were the most common source of infection in the immunocompetent group, whereas bacterial infections were the most common in the immunocompromised groups, even in patients who only had lymphopenia without PLE, further highlighting the complexities of the immune abnormalities seen in Fontan patients even before they are subjected to post-transplant immunosuppressives.^34^

### CAV

CAV is characterized by diffuse, eccentric intimal thickening of the coronary arteries and is thought to be due to a multitude of factors, including chronic inflammation driven by cellular and antibody-mediated rejection as well as other metabolic, genetic, and infectious factors.^37^, ^38^ Once diagnosed, it is a progressive disease process and is the most common cause of graft failure that accounts for about 50% of the need for retransplant in the pediatric population.^38^

Few studies have examined CAV specifically in CHD or SVD patients, but in last year’s PHTS registry analysis, the 10-year freedom from the development of CAV has only slightly improved over the three eras, with 74% freedom from CAV in era one compared to 80% in the most recent era.^13^ Unfortunately, both the International Society for Heart and Lung Transplant and PHTS registries report that survival following diagnosis of CAV remains poor, with only about 70%-75% survival at 5 years and no significant improvement over the decades. 13, 39 There is no difference in the freedom from CAV between patients with cardiomyopathy, CHD-BVD, and CHD-SVD.^13^, ^39^ Within the Fontan population, there is no difference in the time to CAV diagnosis between patients with a competent immune system versus patients with lymphopenia and/or PLE. The causes of CAV continue to remain elusive but do not seem to be modified by the pre-transplant presence of CHD.

### Renal disease

Renal dysfunction following heart transplant is typically a result of nephrotoxicity from chronic calcineurin inhibitor needs and has a low but important risk of progression to end-stage renal disease (ESRD) requiring hemodialysis or renal transplant. There are no studies looking specifically at the development of renal disease in the single ventricle population following heart transplant; however, some inferences may be made based on data and knowledge from other reports. For example, glomerular and tubular abnormalities are a well-studied consequence of long-standing cyanotic CHD, thought to be due to the effects of hypoxia and resultant polycythemia on the renal vasculature and tissue.^40^ Additionally, a pre-transplant diagnosis of CHD as well as RHSC within the first year following transplant have been reported as potential risk factors for kidney injury following heart transplant.^41^, ^42^ The combination of possible underlying renal abnormalities along with the higher rates of RSHC following transplant in the SVD population may predispose these patients to developing renal disease after transplant.

mTOR inhibitors (such as everolimus) generally exhibit less nephrotoxicity compared to calcineurin inhibitors and theoretically could help mitigate the risk of renal injury in pediatric heart transplant recipients. The TEAMMATE trial, which is the first multicenter randomized control trial within the field of pediatric heart transplant, compared the current standard maintenance immunosuppression regimen of tacrolimus with mycophenolate mofetil against everolimus with lose-dose tacrolimus to evaluate the development of major adverse transplant events.^43^ The everolimus/low-dose tacrolimus group was reported to have numerically lower rates of renal dysfunction at a follow-up of 30 months post-transplant as well as a higher estimated glomerular filtration rate (although there was no significant difference in the composite major adverse transplant event score between the two treatment groups).^43^ It remains to be seen if the use of mTOR inhibitors as primary immunosuppression with a low-dose calcineurin inhibitor has the same impact on the development of nephropathy in single ventricle transplant patients and if long-term use will decrease the risk of chronic kidney disease and ESRD in post-transplant patients.

### Post-transplant lymphoproliferative disease (PTLD) and malignancy

Malignancies in pediatric heart transplant recipients are relatively uncommon, occurring in about 5%-10% of patients by ten years post-transplant, with a vast majority of the diagnoses being post-transplant lymphoproliferative disease (PTLD).^44^, ^45^ Epstein-Barr virus (EBV) is a primary driver of pathogenesis in PTLD, with numerous studies showing that EBV infection and EBV donor-recipient mismatch are strongly associated with the development of PTLD.^44^, ^45^, ^46^ Over the last three decades, there has been little change in freedom from malignancy, and survival rates following diagnosis have remained consistently low.^13^ Patients with CHD have been found to have higher rates of malignancy compared to those with cardiomyopathy; however, there are very limited data to further classify the risk of malignancy by CHD diagnosis (including SVD versus BVD).^13^ There has been some suggestion in the PHTS study examining the Fontan immunophenotype that there is no correlation between the presence of lymphopenia and/or PLE and the development of PTLD; however, the patient cohort that developed PTLD in this study was very limited.^34^

## Conclusions

Outcomes in pediatric heart transplantation have steadily improved since the first pediatric heart transplant was performed by Dr. Kantrowitz and his team in 1967; however, there is still progress to be made. Patients with CHD consistently have worse waitlist mortality, post-transplant survival, infection rates, and PTLD risk, with SVD patients being at particularly high risk for rejection and RSHC. Furthermore, there has been little improvement in the development of CAV and PTLD over the decades, and survival following diagnosis continues to remain poor for all patients regardless of diagnosis. Hopefully, as new advances in immunosuppression, mechanical support, and surgical techniques emerge, we will continue to develop a deeper understanding of the co-morbidities associated with pediatric heart transplantation to further improve the survival and quality of life of pediatric heart transplant recipients.

## Disclosures

The authors have no funding sources or conflicts of interest to disclose.

## Declaration of Competing Interest

The authors declare that they have no known competing financial interests or personal relationships that could have appeared to influence the work reported in this paper.
